# Supplementary material for: Content-rich biological network constructed by mining PubMed abstracts
Source: BMC Bioinformatics. 2004 Oct 8;5:147. doi: 10.1186/1471-2105-5-147 (PMC528731; doi:10.1186/1471-2105-5-147)
Supplement: Additional File 5 — The original Chilibot query results of the term "long-term potentiation (LTP)" and 22 other terms, limiting the latest references analyzed to the years 1990, 1995, 2000, and 2004. [file 1471-2105-5-147-S5.bz2 › chilibotAdditionalFile5/ltp2004/html/NMDA_CAMKII.html]

 


 **NMDA** and **CAMKII** 
  
Found 99 abstracts in PubMed,  **30 abstracts were retrieved and analyzed**.  


---

 Search Google  |
 PDF files only 
|  EDU domain only 

---

**Interactive relationship** (e.g. stimulation, inhibition, etc)

- Metabolic labeling of hippocampal cultures reveals that N methyl D aspartic acid  **NMDA**  receptor activation induces  **CaMKII**  dependent phosphorylation of SAP97.  Ref: 12933808 J Biol Chem, 2003
- These data strongly suggest that  **NMDA**  receptor channels mediated the calcium dependent activation of  **CaMKII**  and  **NMDA**  receptors surely were the substrates on membranes of active  **CaMKII** .  Ref: 12650977 Brain Res, 2003
- Taken together, these results suggest that the facilitating action of clozapine on the  **NMDA**  and electrically evoked responses in pyramidal cells of the mPFC requires activation of  **CaMKII**  enzyme.  Ref: 12539202 Synapse, 2003
- In particular, the central participation of  **CaMKII**  as a sensor of the calcium signals generated by activation of  **NMDA**  receptors after the induction of long term plastic changes, has encouraged the use of pharmacological, genetic, biochemical, and imaging tools to unveil the role of this kinase in the acquisition, consolidation, and expression of different types of memories.  Ref: 12469869 Cell Mol Neurobiol, 2002
- ORL 1 receptor knockout mice exhibited a hyperfunction of N methyl D aspartate  **NMDA**  receptor, as evidenced by an increase in 3 H MK 801 binding,  **NMDA**  evoked 45 calcium uptake and activation of calcium calmodulin dependent protein kinase II  **CaMKII**  activity and its phosphorylation as compared with those in wild type mice.  Ref: 12888804 Mol Psychiatry, 2003
- Treatment of cultured hippocampal neurons with  **NMDA**  for 2 min promotes a 2.3 fold thickening of the PSD and a 4 fold increase in PSD associated  **CaMKII**  immunolabel.  Ref: 14501202 J NeurocytolJ Neurocytol, 2003
- The D4 modulation of  **NMDA**  receptors in PFC involved the inhibition of protein kinase A, activation of protein phosphatase 1 and the ensuing inhibition of active calcium calmodulin dependent kinase II  **CaMKII** .  Ref: 14586014 J Neurosci, 2003
- conversely, autophosphorylated  **CaMKII**  could modulate  **NMDA**  receptor channel properties by phosphorylating NR2B.  Ref: 12650977 Brain Res, 2003
- The  **NMDA**  receptor also serves as a  **CaMKII**  docking site in dendritic spines with high affinity binding sites located on its NR1 and NR2B subunits.  Ref: 12379661 J Biol Chem, 2002
- Our findings suggest that SAP97 NR2A interaction is regulated by  **CaMKII**  dependent phosphorylation and provide a novel mechanism for the regulation of synaptic targeting of  **NMDA**  receptor subunits.  Ref: 12933808 J Biol Chem, 2003
- All these data imply the close relationships between  **CaMKII**  and NR2B during ischemia and reperfusion, namely,  **CaMKII**  might act as an amplifier of detrimental cellular calcium signal regulated by  **NMDA**  receptors when becoming autophosphorylated and targeting to NR2B.  Ref: 12650977 Brain Res, 2003
- When the phosphatase inhibitors, calyculin A or okadaic acid, are included in the medium, the  **NMDA**  induced thickening of the PSD as well as the increase in PSD associated  **CaMKII**  immunolabeling are largely maintained 75% and 88% of the peak values respectively at 5 min after removal of  **NMDA**  and calcium from the medium.  Ref: 14501202 J NeurocytolJ Neurocytol, 2003
- Together with previous studies, these results indicate that calcium influx elicited by active  **NMDA**  receptors and L VGCC triggers the Pyk2 Src signaling pathway mediated by PKC, which aggravates cerebral ischemia lesions through up regulating the function of  **NMDA**  receptors after the onset of ischemia, and also could be regulated partly by CaM dependent kinases like  **CaMKII** .  Ref: 14732460 Neurosci Lett, 2004
- The regulation of  **CaMKII**  levels uses distinguishable pathways, one responsive to  **NMDA**  receptor blockade that controls alphaCaMKII alone, the other responsive to AMPA receptor blockade and involving betaCaMKII and possibly further downstream effects of betaCaMKII on alphaCaMKII.  Ref: 12495625 Neuron, 2002
- **NMDA**  stimulation of phospho PKB was blocked by wortmannin, the  **CaMKII**  inhibitor KN 93, MK 801, and removal of extracellular calcium.  Ref: 12358757 J Neurochem, 2002

**Parallel relationship** (e.g. studied together, co-existance, homology, etc.)

- Interactions with various subunits of the  **NMDA**  subtype of glutamate receptor have attracted the most attention, but binding of  **CaMKII**  to cytoskeletal and several other regulatory proteins has also been reported.  Ref: 14653781 Biochem JBiochem J, 2002
- Because AMPA and  **NMDA**  receptors, as well as l type calcium channels are calcium permeable, we also investigated the role of the calcium activated second messenger calcium calmodulin dependent protein kinase II  [ **CAMKII** ]  CaM KII.  Ref: 14725635 Eur J NeurosciEur J Neurosci, 2002
- Using the method of intracellular recording in in vitro brain slices, we investigated whether calcium calmodulin dependent kinase II  **CaMKII**  is involved in the facilitating action produced by the atypical antipsychotic drug APD clozapine on N methyl D aspartate  **NMDA**  induced inward currents and electrically evoked excitatory postsynaptic currents EPSCs in pyramidal cells of the medial prefrontal cortex mPFC.  Ref: 12539202 Synapse, 2003
- To further illustrate the mechanisms underlying these processes, we examined the effects of ketamine a selective antagonist of  **NMDA**  receptor, KN 62 1 N, O bis 5 isoquinolinesulfonyl N methyl L tyrosyl 4 phenylpiperazine, a selective inhibitor of  **CaMKII**  and reperfusion on  **CaMKII**  and  **NMDA**  receptors and the interactions between these signal proteins.  Ref: 12650977 Brain Res, 2003
- In contrast to results in pyramidal cells from rats or wild type mice, clozapine was not able to potentiate  **NMDA**  induced currents in the mPFC pyramidal cells from the  **CaMKII**  mutant mouse.  Ref: 12539202 Synapse, 2003
- In addition, following  **NMDA**  treatment, PSDs exhibit a 7.5 fold increase in labeling with an antibody specific to the Thr286 phospho form of  **CaMKII** , indicating that  **CaMKII**  translocated to the PSD is phosphorylated.  Ref: 14501202 J NeurocytolJ Neurocytol, 2003
- We also found that H 2 O 2 induced apoptotic like cell death was partially prevented by elimination of extracellular calcium, or by inhibition of  **NMDA**  R, L VGCC, PTK and  **CaMKII** , respectively.  Ref: 14707549 J Neurocytol, 2003
- We show that Ras relays the  **NMDA**  R and  **CaMKII**  signaling that drives synaptic delivery of AMPA Rs during long term potentiation.  Ref: 12202034 Cell, 2002
- The role of  **NMDA**  glutamate receptors, PKA, MAPK, and  **CAMKII**  in the hippocampus in extinction of conditioned fear.  Ref: 12625457 Hippocampus, 2003
- The neurotoxic event is characterized by increased alpha  **CaMKII**  association to NR2 regulatory subunits of  **NMDA**  receptor complex.  Ref: 12372013 Eur J Neurosci, 2002
- The  **NMDA**  induced  **CaMKII**  activation in the hippocampal slices of wild type mice was significantly inhibited by exogenous nociceptin via a pertussis toxin sensitive pathway.  Ref: 12888804 Mol Psychiatry, 2003
- The glutamate mediated increase in association of p35 and CaMKIIalpha is mediated in large part by  **NMDA**  receptors, suggesting that cross talk between the Cdk5 and  **CaMKII**  signal transduction pathways may be a component of the complex molecular mechanisms contributing to synaptic plasticity, memory, and learning.  Ref: 12223541 J Neurosci, 2002
- The  **CaMKII**  inhibitor, KN 93 N 2 N 4 Chlorocinnamyl N methylaminomethyl phenyl N 2 hydroxyethyl 4 methoxybenzenesulfonamide... KN 92 2 N 4 Methoxybenzenesulfonyl amino N 4 chlorocinnamyl N methylbenzylamine, phosphate, blocked clozapine s augmenting effect on  **NMDA**  evoked responses in pyramidal cells of the rat mPFC.  Ref: 12539202 Synapse, 2003
- We conclude that the NR1 C0 region is a key site for recruiting  **CaMKII**  to the postsynaptic site, where it may act in concert with calmodulin to modulate the stimulatory role of alpha actinin interaction with the  **NMDA**  receptor.  Ref: 12379661 J Biol Chem, 2002
